# Estimation of Pap-test coverage in an area with an organised screening program: challenges for survey methods

**DOI:** 10.1186/1472-6963-6-36

**Published:** 2006-03-17

**Authors:** Paolo Giorgi Rossi, Gennaro Esposito, Silvia Brezzi, Angela Brachini, Patrizio Raggi, Antonio Federici

**Affiliations:** 1 Agency for Public Health, Lazio Region, Rome Italy. Via di S. Costanza, 53, 00198, Rome, Italy; 2 Viterbo Local Health Unit, Department of Prevention. Viale Trento 40, Viterbo 10100, Italy; 3 Ospedale Belcolle, Strada Sammarinese, Viterbo 10100, Italy

## Abstract

**Background:**

The cytological screening programme of Viterbo has completed the second round of invitations to the entire target population (age 25–64). From a public health perspective, it is important to know the Pap-test coverage rate and the use of opportunistic screening. The most commonly used study design is the survey, but the validity of self-reports and the assumptions made about non respondents are often questioned.

**Methods:**

From the target population, 940 women were sampled, and responded to a telephone interview about Pap-test utilisation. The answers were compared with the screening program registry; comparing the dates of Pap-tests reported by both sources. Sensitivity analyses were performed for coverage over a 36-month period, according to various assumptions regarding non respondents.

**Results:**

The response rate was 68%. The coverage over 36 months was 86.4% if we assume that non respondents had the same coverage as respondents, 66% if we assume they were not covered at all, and 74.6% if we adjust for screening compliance in the non respondents. The sensitivity and specificity of the question, "have you ever had a Pap test with the screening programme" were 84.5% and 82.2% respectively. The test dates reported in the interview tended to be more recent than those reported in the registry, but 68% were within 12 months of each other.

**Conclusion:**

Surveys are useful tools to understand the effectiveness of a screening programme and women's self-report was sufficiently reliable in our setting, but the coverage estimates were strongly influenced by the assumptions we made regarding non respondents.

## Background

The Pap-test is an effective preventive measure in reducing cervical cancer incidence and mortality [1]. It is recommended by the EU commission guidelines for all women aged 25–64 once every 3–5 years [2]. These guidelines also recommend implementing organised screening programs, because they assure quality control, timeliness of testing, and equity of access [1,3].

Italian law requires the operation of screening programmes in every health district, but some have yet to be implemented [4]. However, even when a programme is successful at contacting the entire target population, compliance varies from 20% to 60%, with a mean of 39% (5, 6).

There are several indicators suggesting that most of the target population undergoes opportunistic Pap-testing and that the total coverage of the population varies from area to area. For example, the total annual number of Pap-tests performed by opportunistic screening in Italy is about 5.000.000, i.e. enough to cover the Italian target population of 16.000.000 every three years; but evidence from the national health survey shows that coverage ranges from 34% to 78% in the different regions of Italy [7].

In addition to knowing the coverage of the target population, it is important to identify the use of opportunistic screening both in women who are compliant and those who are not compliant to screening programs: in fact, addressing inappropriate use of resources is essential to build an efficient and equitable health service.

For these objectives, the most used study design is the survey and the most common method used is a telephone interview [7,8]. Several authors have questioned the validity of surveys, and in particular two problems have been highlighted: the validity of recall [9-24] and the assumption about non respondents [25-30].

### Objectives of the study

The goals of the survey were to:

• Estimate the Pap-test coverage in the area of Viterbo where an organised screening program is active.

• Identify the reasons for non compliance to the screening invitation.

We addressed the following methodological issues in analysing these data:

• We assessed the validity of the women's recall.

• We propose a sensitivity analysis on the assumptions about the non respondents.

• We developed a method to estimate the role of address accuracy in screening compliance.

## Methods

### The setting

The Viterbo Health District is a rural area in central Italy with 291.000 inhabitants (target population 81.000 25–64 year old women). The screening program in Viterbo started in 1997 and the second round finished in the summer of 2004. Every three years it invites all female residents aged 25–64 in the area to be screened. The average compliance is 42%. All mailings, reminders, Pap-tests, colposcopies, histological samples and treatments are recorded individually in the program database.

### Study design

**Figure 1:**
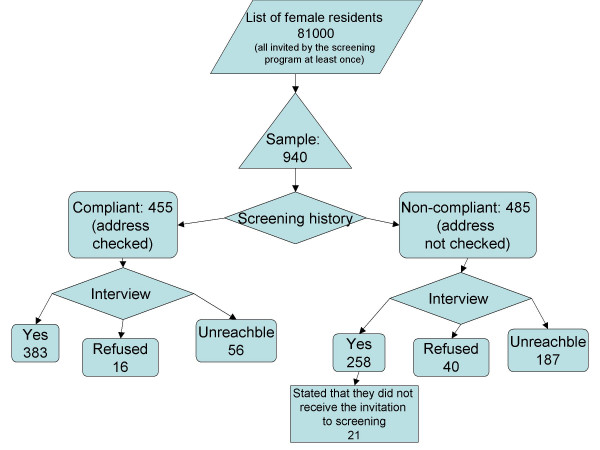
Study flow chart.

This is a study integrating data from a cross-sectional survey and from the screening program registry (figure 1). We sampled 940 women aged 25–64, from the list of residents in the Health District, in two strata: residents of the city of Viterbo and residents of the rest of the district.

The sample size was determined to have a power of 80%, with alpha 0.05, to detect a 10% difference in coverage between the two population groups, with a mean prevalence of 50%, the smallest group representing at least 30% of the sample. The resulting sample size is 600; the expected response rate was 65%. The resulting precision for a point estimate of a hypothetical 50% coverage is +/- 2%.

### The questionnaire

The questionnaire included the following sections: individual data (educational level, occupation, employment); hysterectomy; family history of cancer; screening history (when and where of last Pap-test; test frequency); reasons for not complying with screening program; satisfaction with service. The phone questionnaire took about 15 minutes, and was conducted by three trained interviewers. The survey was conducted between May and October 2004. All women sampled were phoned up to 9 times, then a reminder was mailed to the women who had not yet been contacted, after which another 3 phone contacts were made. The phone calls were made at different hours of the day.

### Analysis

The information collected in the interview about Pap-tests performed by the screening program was compared with the data in the screening archives.

We used an algorithm to estimate the proportion of women unreachable for screening, (31) based on the following assumptions: 1) that we have the correct addresses and phone numbers of the women who have been compliant to screening at least once, but may not for women who were never screened; 2) that the difference between the proportion of not contacted women among the non-compliant women and the proportion of not contacted women among the compliant is due to the wrong addresses (and consequently phone numbers) in the non-compliant women; 3) that the women are honest when stating they have received the screening invitation letter; 4) and that the entire sampled population has been invited at least once. The resulting algorithm for the proportion of unreachable women is: (% of women who never received the letter)+((% of women not contacted in the group of the non compliant to screening)-(% not contacted in the screening compliant group)).

We performed a sensitivity analysis for 36-month coverage and used three estimates according to different hypotheses on non-respondents:

1. We considered crude coverage (hypothesis: non-respondents = respondents), the least conservative hypothesis;

2. The intermediate hypothesis adjusted the coverage of non-respondents by screening compliance: coverage among non-respondents to the interview = (observed coverage among screening non-compliant)*(proportion of non-compliant among non-respondents)+(observed coverage among screening compliant)*(proportion of non-compliant among non-respondents);

3. In the third and most conservative hypothesis only the compliant are considered covered, so all non-respondents who were not compliant to screening are considered not covered.

### Ethics and privacy

The Regional Ethics Committee stated that the Screening Program of each Health District must provide a Pap-test every three years to all residents in the target population, and this task involves the ascertainment of actual coverage.

The interviews were anonymous, only a personal identification number, internal to the Screening Program database, permitted the linkage with screening history records.

## Results

### Response rate

We interviewed 641 women out of 940 sampled (68.2%), 6% refused to participate and 25.9% were unreachable. We did not find any difference in the response rate by age or residence.

We sampled 455 women who had had at least one Pap-test in the screening program and 485 who never complied with screening. We observed a highly significant difference in response rate between the screening compliant and the non-compliant: we interviewed 84.2% of the screening compliant women and 53.2% of the non compliant; the difference was mostly due to the unreachable women (12.3% vs. 38.6%), rather than to refusals (3.5% vs. 8.3%).

### Pap-test coverage

We excluded from the analysis 22 (3.4%) women who had had a hysterectomy. Fifty-nine declared to never have had a Pap-test in their life, the crude coverage once in a lifetime is 90.1% (95%CI 87.3–92.8); only one woman stated that she did not know what a Pap-test is. Eighty-six point four percent (95%CI 83.3 – 89.5) reported having had a Pap within 36 months, and 88.5% (95%CI 85.6 – 91.4) within 60 months. We asked the compliant women what their Pap-test history was at their first appointment with the screening program, and 217 women answered: 18% (40) never had a Pap-test previously, only 5 of them were 25 years old at the time of the first invitation; 32% (70) had a Pap-test more than three years before.

**Figure 2:**
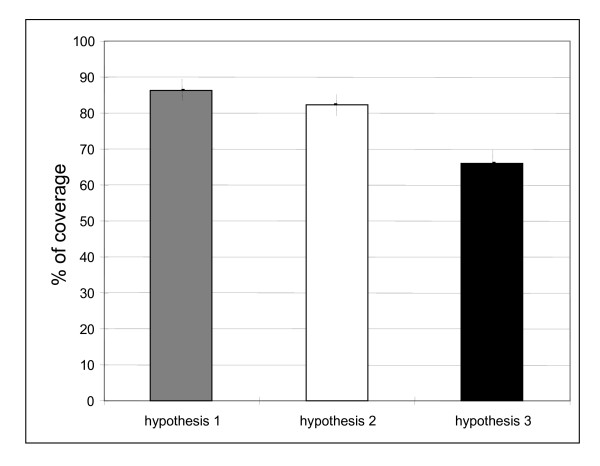
Results of the sensitivity analysis for 36-month coverage estimates. Hypothesis 1: crude coverage (hypothesis non-respondents = respondents); hypothesis 2: coverage in the non-respondents adjusted by screening compliance, i.e. coverage among non-respondents = (observed coverage among screening non-compliant)*(proportion of non-compliant among non-respondents)+(observed coverage among screening compliant)*(proportion of non-compliant among non-respondents); hypothesis 3: all the non-respondents non-compliant to screening are not covered and only the compliant are covered. The bars represent the 95% confidence interval.

We performed a sensitivity analysis of coverage at 36-months according to our different hypotheses on the coverage among non-respondent women. The coverage among screening compliant was 92.4%, among non-compliant 69.0%; after adjusting for screening compliance the predicted coverage among non-respondents was 74.6% and 82.4% in the general population; according to the most conservative hypothesis, i.e. none of the non compliant who did not respond were covered, the estimate is 66.0% (figure 2).

The multivariate analysis showed a strong association with age, (women under 30 years old were less likely to be covered) and a weak association with having more than 5 years of education. We did not observe an association with residence.

Tables 1 and 2 show the distribution of the answers to the question "when did you have your last Pap-test" and "how frequently do you have Pap-tests", by type of provider. Women who were last tested by a public provider tended to have a bimodal distribution with peaks at one and at three year periods (the periods used by the screening programme and recommended by the National Health Service Guidelines), while the women who had their last Pap-test in a private clinic had a single peak at one year.

### Reasons for non compliance to screening invitation

The first reason for non compliance is not receiving the letter. We estimated the proportion of unreachable women, using the algorithm described in the methods: the not-reached proportion of the non compliant (38.6%), minus the not-reached proportion of the compliant (12.3%), plus those who reported not receiving the letter (9%). About 35% of the not compliant were not screened because they did not receive the invitation, corresponding to 20% of the general population.

Table 3 summarises the self-reported reasons for non compliance to screening invitation, by last Pap-test: women who never had a Pap reported "fear" as the main reason, while women who had had a Pap in the private sector reported "no trust in public services" or "exclusive trust in private gynaecologist".

Just over half of the interviewed women who had had at least one Pap-test were tested most recently by the screening programme, 10.9% in another public clinic and 38.0% in a private clinic. We asked all women who had had their most recent Pap outside the screening program if they had ever had a Pap in the screening programme: 30% of them also had had a Pap in the screening program, a proportion that was higher in women who visit public clinics (43%).

**Figure 3:**
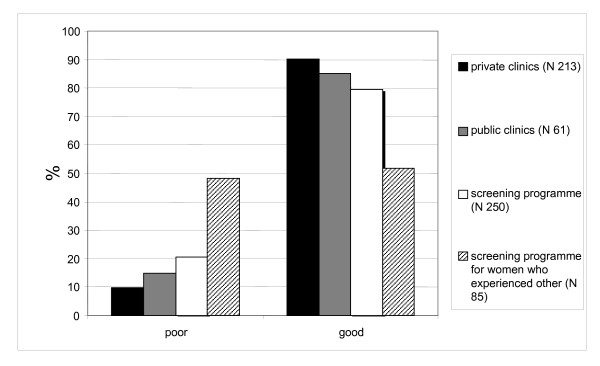
Women's satisfaction according to the Pap-test provider.

We measured the women's satisfaction with the test procedures (figure 3). Women who had the Pap in a private clinic were significantly more satisfied than women who were screened (90.0% vs 79.6%, chi2 = 9.7; p = 0.002), particularly those who had been tested in both places (90.0% vs. 51.7%, chi2 = 24.7: p < 0.00005). We did not observe differences in satisfaction by age, residence or educational level.

### Validity of self-report (recall)

We compared the information from the screening registry with the answers given to the interview. Table 4 shows the sensitivity (84.5%; 95%CI 80.9–88.3) and specificity (82.2%; 95%CI 76.9–86.6) of the question "ever had a Pap-test in the screening program".

**Figure 4:**
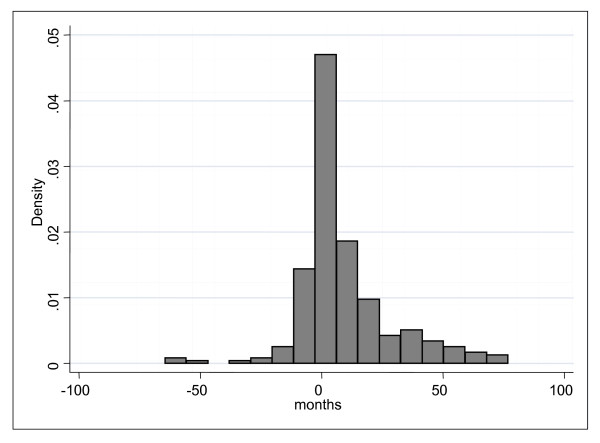
Frequency distribution of the difference between the self reported Pap-test dates and the dates reported in the screening registry. A positive value indicates that the woman's reported date was more recent than the registry date. The variable is defined for the 267 women who declared to have had the last Pap in the screening programme and had a Pap recorded in the screening registries.

Figure 4 shows the difference in months between the self-reported Pap-test date and the date reported in the screening registry. The variable was present for the 267 women who reported having had their last Pap in the screening program that was recorded in the screening registries. The mode of the distribution was +/- 1 month, 68% of the observations fell between +/- 12 months. There was asymmetry in the distribution: the self reported dates tended to be more recent than the dates in the registry.

## Discussion

Surveys to measure test coverage for cancer prevention have been conducted in several countries [8] to monitor the efficacy of information campaigns and to understand non compliance to screening. In Italy however, although public screening programmes have been activated in almost all parts of the country, the compliance to screening programs is low in many districts. The question is: what is the real Pap-test coverage rate? The National Health Interview gives an estimate of 68% for the country, and 77% for our region [7].

We tried to answer this question with this study, in response to a mandate from the Italian Group for Cytological Screening, but also had to address several methodological issues common to all surveys that try to estimate the coverage achieved by screening tests.

### The coverage

Our estimate for Pap-test coverage once in a lifetime is 90%, while the National Health Interview of 1999–2000 found it to be 77% in our region [7]. This difference is probably due to a real increasing trend in the area, consequent to cultural changes and to the activation of the first round of public screening in 1998–2001 that invited the entire target population and was promoted locally. We know that 50% of the women at their first appointment with the screening program were not adequately covered, consequently, because the compliance to screening was about 42%, the impact of the program showed an increase of at least 20% of the actual coverage.

The coverage estimate changes according to the assumption we used regarding the non respondents. This problem has been addressed in many surveys [25,29,32-34]. In this case, we were fortunate to know the Pap-test status for about 45% of the target population from the screening registries. Combining the information from the survey with that from the screening registry we observed that: 1) the non respondent population is very different from the respondent population regarding their utilisation of screening; 2) the unadjusted results of the survey overestimate Pap-test coverage. For the sensitivity analysis we used three different hypotheses. For the intermediate hypothesis, we adjusted for screening compliance assuming that the non respondent women compliant to screening have the same coverage as the respondents compliant to screening, and that the non-respondent women non-compliant to screening have the same coverage as the respondent non-compliant to screening. This method gives results similar to those from other surveys interviewing only non compliant women, proposed by several authors that was used to estimate the Pap-test coverage in areas where screening programs are active and have a registry available [35,36].

The provider and the periodicity were associated: women who had their last Pap in a private clinic repeated Pap-tests more frequently than women who were last tested by the screening program. Given the cross-sectional design we cannot affirm that the provider influences the women's behaviour; it could be a self selection effect: women who want more Pap-tests go to private clinics because the screening programme only provides one Pap every three years. Nevertheless, although we cannot say that the screening program decreases over-screening, we can affirm that it is associated with more appropriate timing.

### Reasons for non compliance

The major barrier to screening was the low reliability of the address list for mailing the invitations, in fact, we estimated that 21% of the women could not be reached.

The algorithm we used to estimate the proportion of women who were unreachable is simple and is based on reasonable, although not perfect assumptions. This method however, in our opinion, is useful since there are no other simple solutions to assess the quality of the address lists.

The second reason for non compliance is the lack of trust in public service, an old problem in some areas of central and southern Italy.

Even though 75% of women were satisfied with the screening programme, it is significantly worse than for private providers; particularly concerning is the low satisfaction with screening in women who had been both privately tested and screened. We have no indications about the reasons for the dissatisfaction, but it may be intrinsic to the organisation of our and other mass screening programs where the physician does not reassure the patients [37] during the sampling, and negative results are communicated only by a mail, about one month later.

### Methodological remarks and limits

The validity of self-reported information about Pap-tests has been studied in several surveys, which have reached different conclusions. It is evident from most of these studies that the validity of women's recall varies according to the socio-economic and cultural environment, so the results change according to geographical area, ethnicity, type of health care provider, and even neighbourhood [9,10,13,15-17]. Most of the literature about this topic has been published in the US, in order to validate the results of the NHIS [38] and other surveys [39] used to measure the progress in cancer prevention and the achievement of the National Cancer Institute goals. Few European studies have been published [40], and none in Italy recently (36, 41–43). In our population the validity of self-reported Pap-tests is high and only slightly overestimated coverage, because the sensitivity and the specificity are similar. The effect of telescoping [44], the tendency to report a test as having occurred more recently than it actually did, is modest, nevertheless it must be taken into account that when the source of information to measure three years coverage is women's recall, what is actually being measured is the percentage of women who had a Pap-test in the last 30–48 months. The district of Viterbo is mainly rural, with a pro-capita yearly income close to the Italian mean (24,000 €), the educational level of the population is similar (9% did not complete middle school and 31% have a high school degree; the national figures are 10% and 33%, data from the National Institute of Statistics – ISTAT), and it has an immigrant population of 3.4% (the Italian average is 3.5%). This picture of the study population is consistent with the results about validity [18,45]. The major, and unavoidable, limit of our study for validating the self-reports is that we can only validate the tests performed within the screening programme, not those performed privately or in other settings that have no registries, nevertheless we do not see any reason for which women should preferentially forget the tests performed in a screening program or in another provider.

## Conclusion

Surveys are a useful tool to determine the effectiveness of a screening program in maintaining high Pap-test coverage, but the coverage estimates we can obtain are strongly influenced by the assumptions we make about non-respondents. Adjusting for screening compliance among non respondents give a more sound estimate than assuming equal coverage in respondents and non respondents.

In our setting the most important reason for non compliance to screening is that we were unable to contact 20% of the target population: every effort possible should be made to obtain reliable address lists.

## List of abbreviations

EU European Union

CI confidence interval

## Competing interests

The author(s) declare that they have no competing interests.

## Authors' contributions

PGR conceived and designed the study, conducted the statistical analysis and wrote the paper. RE also conceived and designed the study, collected data, helped with the statistical analysis and writing the paper. AB was involved in designing the study and in data collection. SB conceived and designed the study, and helped writing the paper. AF was involved in conceiving the study and writing the paper. All authors read and approved the final manuscript.

## Pre-publication history

The pre-publication history for this paper can be accessed here:



## Figures and Tables

**Table 1:** Test periodicity. How frequently the women declared to repeat Pap-test in months, by type of provider.

	Private		Public	
		
Months	N	%	N	%
3	0	0,0	1	0,3
6	8	3,8	2	0,6
12	129	60,6	97	28,0
15	1	0,5	2	0,6
18	6	2,8	6	1,7
24	39	18,3	64	18,4
36	24	11,3	168	48,4
48	2	0,9	1	0,3
60	1	0,5	3	0,9
72	1	0,5	2	0,6
84	1	0,5	1	0,3
120	1	0,5	0	0,0

Total	213	100,0	347	100,0

**Table 2:** Self reported time elapsed from the last Pap-test in months, by type of provider.

	Private		Public	
		
Months	N	%	N	%
0–12	154	72,6	231	66,6
13–24	40	18,9	88	25,4
19–24	32	15,1	73	21,0
25–36	12	5,7	11	3,2
37–48	2	0,9	6	1,7
49–60	2	0,9	5	1,4
61+	2	0,9	6	1,7

Total	212	100,0	347	100,0

**Table 3:** Self-reported reasons for non compliance to screening invitation, by provider of last Pap-test.

	private		public		never screened	
			
reasons for non compliance	N	%	N	%	N	%
did not receive the letter	21	9,9	8	13,1	10	16,9
Forgot	40	18,8	27	44,3	22	37,3
pregnancy	10	4,7	1	1,6		
no trust in public health services	13	6,1			1	1,7
exclusive trust in private gynaecologist	62	29,1				
Fear	8	3,8			16	27,1
did not consider important					5	8,5
hysterectomized					1	1,7
Illness					1	1,7
Virgin					3	5,1
do not respond	59	27,7	25	41,0		

Total	213	100,0	61		59	100,0

**Table 4:** Sensitivity and specificity of the phone interview question "Have you ever had a Pap-test performed by the public screening programme?", according to the screening registry.

	screening registry		
	
phone interview	yes	no	total
yes	325	46	371
no	58	212	270

total	383	258	641

	sensitivity	specificity	
	84.5%	82.2%	
